# Distribution of nanoparticles throughout the cerebral cortex of rodents and non-human primates: Implications for gene and drug therapy

**DOI:** 10.3389/fnana.2014.00009

**Published:** 2014-03-17

**Authors:** Ernesto A. Salegio, Hillary Streeter, Nikhil Dube, Piotr Hadaczek, Lluis Samaranch, Adrian P. Kells, Waldy San Sebastian, Yuying Zhai, John Bringas, Ting Xu, John Forsayeth, Krystof S. Bankiewicz

**Affiliations:** ^1^Department of Neurological Surgery, University of California at San FranciscoSan Francisco, CA, USA; ^2^Department of Materials Science & Engineering, University of California at BerkeleyBerkeley, CA, USA

**Keywords:** gene delivery, AAV, liposomes, thalamo-cortico, perivascular, CSF

## Abstract

When nanoparticles/proteins are infused into the brain, they are often transported to distal sites in a manner that is dependent both on the characteristics of the infusate and the region targeted. We have previously shown that adeno-associated virus (AAV) is disseminated within the brain by perivascular flow and also by axonal transport. Perivascular distribution usually does not depend strongly on the nature of the infusate. Many proteins, neutral liposomes and AAV particles distribute equally well by this route when infused under pressure into various parenchymal locations. In contrast, axonal transport requires receptor-mediated uptake of AAV by neurons and engagement with specific transport mechanisms previously demonstrated for other neurotropic viruses. Cerebrospinal fluid (CSF) represents yet another way in which brain anatomy may be exploited to distribute nanoparticles broadly in the central nervous system. In this study, we assessed the distribution and perivascular transport of nanoparticles of different sizes delivered into the parenchyma of rodents and CSF in non-human primates.

## INTRODUCTION

The movement of macromolecules and nanoparticles within the brain is not a simple process and no single mechanism is entirely responsible for the dissemination of such substances. Three main routes of distribution have been described: rapid perivascular flow, rapid paravascular flow of cerebrospinal fluid (CSF), and slow axonal transport of viral particles both anterograde and retrograde. With tools at hand today, we can isolate and interrogate each pathway substantially independently of the others.

Our initial forays into this area arose from investigations into the therapeutic utility of adeno-associated viral (AAV) vector for the treatment of Parkinson’s disease (Bankiewicz et al., 2000). It was soon apparent that pressurized convection-enhanced delivery (CED) infusions of AAV2-hAADC into the primate striatum resulted in pronounced transgene expression in the globus pallidus. At the time, we assumed that this was due to a perivascular mechanism, and subsequent studies have confirmed that this is indeed the case. We showed in rat experiments that perivascular distribution is greatly influenced by heart rate and is extremely rapid (Hadaczek et al., 2006). Infusion of AAV particles or liposomes into rat striatum revealed rapid movement along perivascular tracts leading from the site of infusion into the globus pallidus. This kind of acute distribution is vectorial. Other parts of the basal ganglia, such as substantia nigra, were not the recipient of this type of rapid transport, suggesting that specific vascular tracts are required to enable this process. More recent studies of parenchymal infusion of liposomes visible by real-time magnetic resonance imaging (MRI) have revealed that rapid convective flow is the primary means by which parenchymal infusions can cover volumes of tissue several fold greater than the volume of infusate (Krauze et al., 2005a, 2006, 2009).

In contrast to this rapid distribution of a variety of infusates, the axonal transport of AAV particles occurs over a more extended timeframe. Although AAV particles can indeed be distributed perivascularly in anatomically restricted domains, it is the ability of AAV to be taken up by neurons and transported over long distances that has excited intense interest. We showed some years ago that AAV2 is transported intact from primate thalamus to cortex where release of AAV2 particles results in widespread transduction of neurons in a number of cortical layers to which thalamic neurons project (Kells et al., 2009). Similarly, we described anterograde axonal transport of AAV2 in basal ganglia in primates (Kells et al., 2012) and in rodents (Ciesielska et al., 2011). Anterograde transport is not ubiquitous among AAV serotypes. For example, AAV6 is transported in a retrograde direction and we are actively exploring the repertoire of available serotypes in this regard.

More recently, we have explored CSF infusions as a way to distribute nanoparticles throughout the primate brain (Samaranch et al., 2013). Both AAV7 and AAV9, infused via intrathecal injection (lumbar puncture) or into cisterna magna (CM) injection, direct robust transgene expression throughout spinal cord, brainstem, cerebellum, and cortex. As we discuss below, the pattern of transgene expression is consistent with the paravascular flow of CSF through these regions. We found also that this phenomenon is not restricted to AAV7 and AAV9. Fluorescent micelles are also extensively distributed through the paravascular pathways deep into the brain.

Due to the presence of the blood–brain barrier (BBB), distribution of therapeutic agents within the central nervous system (CNS) is a major problem in drug delivery. In this study, given our current focus, we provide a more detailed view on the distribution of nanoparticles within the parenchyma of rodents and CSF of non-human primates (NHPs), as alternative routes for delivering therapeutic agents *in vivo*. In particularly, we demonstrate how nanoparticles of different properties and sizes can be rapidly distributed to remote regions within the brain, either propelled by an exogenous pressurized delivery and/or by the endogenous flow of the CSF. Each mechanism displays unique anatomical targeting properties that may be exploited to therapeutic effect. This emerging repertoire of routes for infusate distribution also carries implications for delivery of other types of therapeutic nanoparticles. It should be noted that, given the size and “simpler” axonal connectivity of the rodent’s brain relative to that of primates, we generally perform parenchymal injections in rodents and then validate CSF delivery the primate.

## MATERIALS AND METHODS

### ANIMALS

To investigate the transport of AAV particles, 12 Sprague–Dawley (SD) rats (~250–350 *g*) received a unilateral infusion into the thalamus of AAV2-green fluorescent protein (GFP) or AAV6-GFP. To examine the perivascular transport of larger particles five SD animals received a single thalamic infusion of fluorescently labeled DiIC^18^-liposomes (1 μM). To determine whether any ultra-structural changes had occurred after intra-parenchymal pressurized delivery, two animals were processed for electron microscopy (EM) and infused with phosphate buffered saline (PBS) alone with one additional animal serving as a non-infused naïve control. Perivascular transport of AAV particles infused into the CSF (cisterna magna) was examined in four NHPs (*Macaca mulatta*) that received a single injection of either AAV7 or AAV9-GFP (*n* = 2/serotype; Samaranch et al., 2013). To evaluate the transport of larger particles in NHP, one NHP received a single injection of Oregon Green (OG) labeled micelles into the CM. No differences in body weight and/or adverse symptoms were observed throughout the study. All procedures were carried out in accordance with the UCSF Institutional Animal Care and Use Committee (IACUC) and the Animal Care and Use Committee (ACUC) at Valley Biosystems Inc.

### INFUSATES

AAV2-GFP (1.3 vg/mL × 10^13^ vg/mL), AAV6-GFP (1.2 vg/mL × 10^13^ vg/mL), AAV7-GFP (2.0 vg/mL × 10^13^ vg/mL), AAV9-GFP (1.8 vg/mL × 10^13^ vg/mL) were manufactured by the Research Vector Core facility at Children’s Hospital of Philadelphia (~20–25 nm in size; Matsushita et al., 1998). Liposomes were fluorescently labeled with membrane-bound DiIC^18^ 1,1′-dioctadecyl-3,3,3′,3′-tetramethylindocarbocyanine perchlorate as described (~65 nm in size; Krauze et al., 2006). 3-helix micelles are self-assembling amphiphilic peptide-polymer conjugates (~15 nm in size; Dong et al., 2012). The micelle-forming amphiphile is denoted as dC18-1CW(P2K)-P750. This amphiphile was labeled with the fluorescent agent, OG, and the product denoted as dC18-1CW(P2K)-OG (Dong et al., 2012). 10 mg dC18-1coi(P2K)-P750 and 1 mg of dC18-1CW(P2K)-OG were dissolved in 0.5 mL of methanol in a glass vial and the solvent was evaporated in vacuum oven for 3 h. The dried film was rehydrated with 2 mL of 25 mM phosphate buffer, pH 7.4, and the solution was stirred for 16 h to allow assembly of OG-conjugated fluorescent 3-helix micelles. The micelle solution was washed to remove any unincorporated OG and concentrated by spin filtration. The concentrate was washed with water and lyophilized to obtain OG labeled 3-helix micelles. Fluorescent micelle solution was prepared by directly dissolving the lyophilized fraction in phosphate buffer. Micelles at 6 mg/mL were used for *in vivo* injections. Fluorescence was visualized at 488 nm on a Zeiss Axiomat microscope.

### INFUSIONS

Rats were anesthetized with isoflurane (Baxter, Deerfield, IL, USA) and placed in a stereotactic frame (David Kopf Instruments, Tujunga, CA, USA). A 1-cm longitudinal incision was made on the skin overlaying the skull and a burr-hole was drilled at the following coordinates for targeting the thalamus (AP: -2.8, ML: +1.6, DV: -5.5 mm). A fused silica (Polymicro Technologies, USA) with a 1 mm stepped cannula (0.1 mm internal diameter) was used to deliver a total volume of 12 μL in experiments investigating AAV2, AAV6 and EM, whereas, animals infused with liposomes received a total volume of 5 μL at a rate of 0.5 μL/min (for all infusates). Total length of infusion procedure lasted either 24 or 10 min, respectively, plus a final 2 min period prior to cannulae retraction to reduce reflux of infusate.

Non-human primate were sedated with ketamine/xylazine and placed in a prone position in a stereotactic frame with the head flexed. A 3 mL syringe was manually guided into the CM until CSF was aspirated into the syringe. Once the correct position of the needle was verified, 2 mL of infusate was infused into the CM at a rate of 0.5 μL/min. The duration of the infusion procedure was approximately 4 min with a 2 min waiting period prior to withdrawal of the needle.

### TISSUE PROCESSING

Animals were subjected to necropsy either at 3 weeks (rats) or 6 weeks (NHP) after vector infusion or 4 h after micelle delivery (NHP) and transcardially perfused with PBS followed by 4% paraformaldehyde (PFA)/PBS. Brains were harvested, post-fixed in 4% PFA/PBS for 24–48 h and cryoprotected in 30% sucrose. All post-fixed brains were cut into 40 μm serial sections that were then processed for immunohistochemistry. Liposome-treated animals were sacrificed 30 min after infusion with an overdose of sodium pentobarbital solution and their brains were immediately frozen in dry-ice cooled isopentane. Every second 40 μm section was collected and viewed with a Zeiss microscope with a filter appropriate for Cy-3 fluorescence.

### ELECTRON MICROSCOPY

Animals used for EM were transcardially perfused with 0.1 M Sodium Cacodylate (pH 7.4) buffer solution and then fixed with a mixture of 2% Glutaraldehyde, 1% PFA and 0.1 M Sodium Cacodylate. PBS-infused animals were used as controls and the thalamus was carefully dissected and embedded in epoxy resin. Tissue was cut into 1 μm thick sections with a diamond knife (1.5 mm wide blade at a 45° angle; Diatome, USA).

### IMMUNOPEROXIDASE STAINING

A polyclonal antibody against GFP (rabbit anti-GFP, www.millipore.com, Cat. #06-896) was used for immuno-detection of the transgene in animals that received AAV infusions. Briefly, sections were washed with PBS (3 min × 5 min), quenched for endogenous peroxidase activity in 1% H_2_O_2_/30% ethanol for 30 min, and then washed briefly in PBS/1% Tween [phosphate buffered saline including Tween (PBST)]. Sections were blocked in Background Sniper^®^ (Biocare Medical, BS966G) for 30 min and incubated overnight at 4°C with anti-GFP antibody (1:400) or anti-CD31 (Abcam mouse anti-CD31, clone JC/70A; 1:200) in Da Vinci^®^ green diluent (Biocare Medical, PD900). After washing in PBST, sections were incubated for 1 h in Mach-3-rabbit-probe (Biocare Medical, RP531L) at room temperature (RT), washed again and incubated in Mach-3-rabbit-HRP polymer (Biocare Medical, RH531L) 1 h at RT. After incubation, sections were washed in PBST and developed with 3,3′-diaminobenzidine (DAB) for 1 min (DAB Peroxidase Substrate Kit, Vector Laboratories). DAB-processed sections were washed in PBS and mounted on frosted slides.

## RESULTS

### AAV SEROTYPES INFLUENCE AXONAL TRANSPORT

**FIGURE 1:**
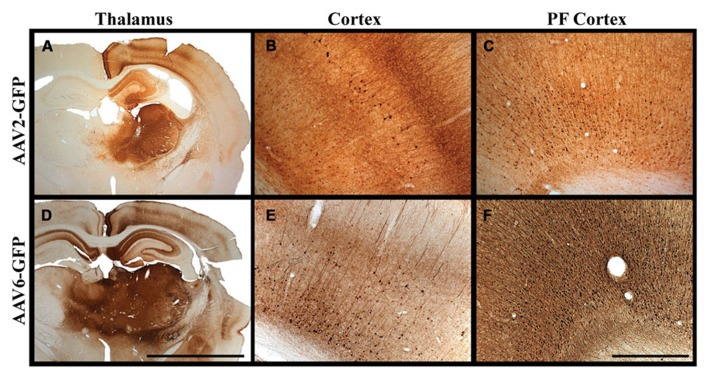
**Transgene distribution after AAV2 and AAV6 delivery. (A–F)** Unilateral injection into the thalamus of adult rats of either AAV2 or AAV6 resulted in widespread expression of GFP transgene in the neocortex and in pre-frontal regions distal to the site of injection. Scale bars: **A,D** = 5 mm; **B,C,E,F** = 1 mm.

The use of AAV vectors encoding fluorescent reporter proteins, such as GFP, permits us to explore anatomical connections *in vivo* by measuring transgene expression at the site of injection and tracking the expression pattern in distal regions. Although axonal transport of viruses is a well-known phenomenon, we were the first to demonstrate that AAV2 not only undergoes anterograde transport but viral particles can apparently be released intact from nerve terminals to transduce other neurons far from the original site of vector infusion in a pattern reflective of known axonal projection pathways. We have demonstrated this in the rodent brain (Ciesielska et al., 2011) and in NHP (Kells et al., 2009). Not all AAV serotypes undergo anterograde transport like AAV2. AAV6, for example, is transported exclusively in a retrograde direction in rodents (Salegio et al., 2013). We initially observed this in a comparison of thalamic infusion of AAV2 with AAV6. In this study, we observed gene transfer throughout all layers of the neocortex (I–VI), including those in pre-frontal regions distal from the site of injection (**Figure 1**). However, the diverse reciprocal connectivity of the thalamus with cortex made definitive analysis difficult. By targeting regions with “simpler” anatomical connections such as the striatum, we could more confidently determine directionality of axonal transport. In rodent striatum, both AAV2 and AAV6 transduce only neurons. AAV2 transduces parts of the basal ganglia to which striatal neurons project, such as substantia nigra pars reticulata (SNr) but does not transduce neurons that project to the striatum such as neurons from the substantia nigra pars compacta (SNc). In contrast, AAV6 transduces SNc, but not SNr, neurons after striatal infusion of AAV6. Moreover, striatal AAV6 transduces cortico-striatal neurons whereas AAV2 does not. This inverse directionality of axonal transport is also seen in NHP with a minor, but potentially significant difference (San Sebastian et al., 2013). AAV6 is not exclusively neurotropic as it is in rodents, although it is a retrogradely transported vector. Although the degree of glial transduction was modest, AAV6-GFP nevertheless triggered a brisk cell-mediated immune response to the non-self transgene, GFP, just as we have seen more intensely with AAV9 (Ciesielska et al., 2013).

Similarly, retrograde transport of adenovirus has been described in rat brain (Kuo et al., 1995). One hypothesis is that differential engagement with molecular machinery that specifies anterograde or retrograde transport may underlie this phenomenon. Herpes simplex virus 1 (HSV-1) interacts with both kinesin and dynein to catalyze anterograde and retrograde axonal transport respectively (Diefenbach et al., 2008). We speculate that these two capacities may be separately defined in structural differences in AAV2 and AAV6 capsids (Gao et al., 2004) and possibly by receptor-cell interactions (Nonnenmacher and Weber, 2012).

### AXONAL VERSUS PERIVASCULAR TRANSPORT

Anterograde axonal transport of AAV2 is a relatively slow process taking at least 6 weeks after delivery to reach an easily detectable level of transgene expression distal from the site of injection (Salegio et al., 2013). However, we have established that AAV particles (~20–25 nm in size), as well as proteins and liposomes (~65 nm in size), can also be distributed very rapidly by non-axonal means. Perivascular transport of various nanoparticles and proteins takes place during convective (pressurized) infusions, the most clear-cut example being distribution of infusates from putamen to globus pallidus. We found that the driving mechanism appears to be arterial pressure (Hadaczek et al., 2006). Increasing blood pressure in rats undergoing striatal infusions significantly increased distribution of infusates and stopping the heart effectively blocked distribution of infusates beyond the local infusion site.

**FIGURE 2:**
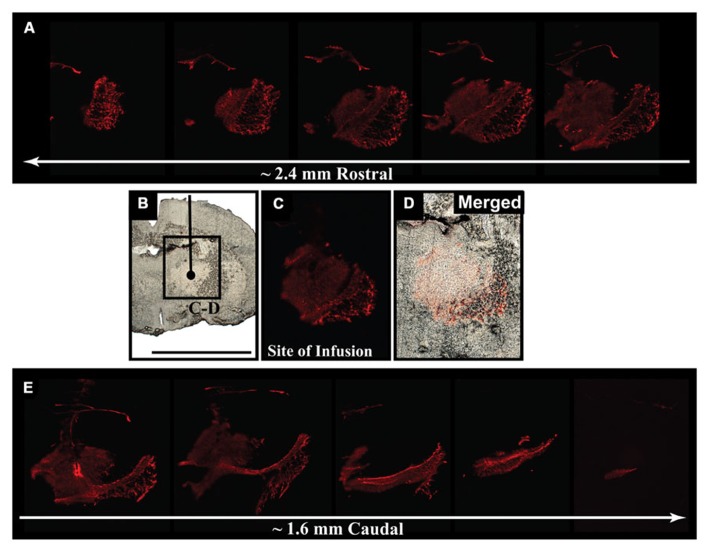
**Early parenchymal distribution of fluorescently labeled liposomes. (A–E)** Acute survival time-point for liposome-infused animals revealed the rapid spread of fluorescently labeled liposomes 30 min after delivery into the thalamus. Spread of fluorescence reached approximately 2.4 mm rostral and 1.6 mm caudal from the site of infusion, suggesting efficient movement of these particles throughout the brain after CED. Scale bar: **B–D** = 5 mm.

**FIGURE 3:**
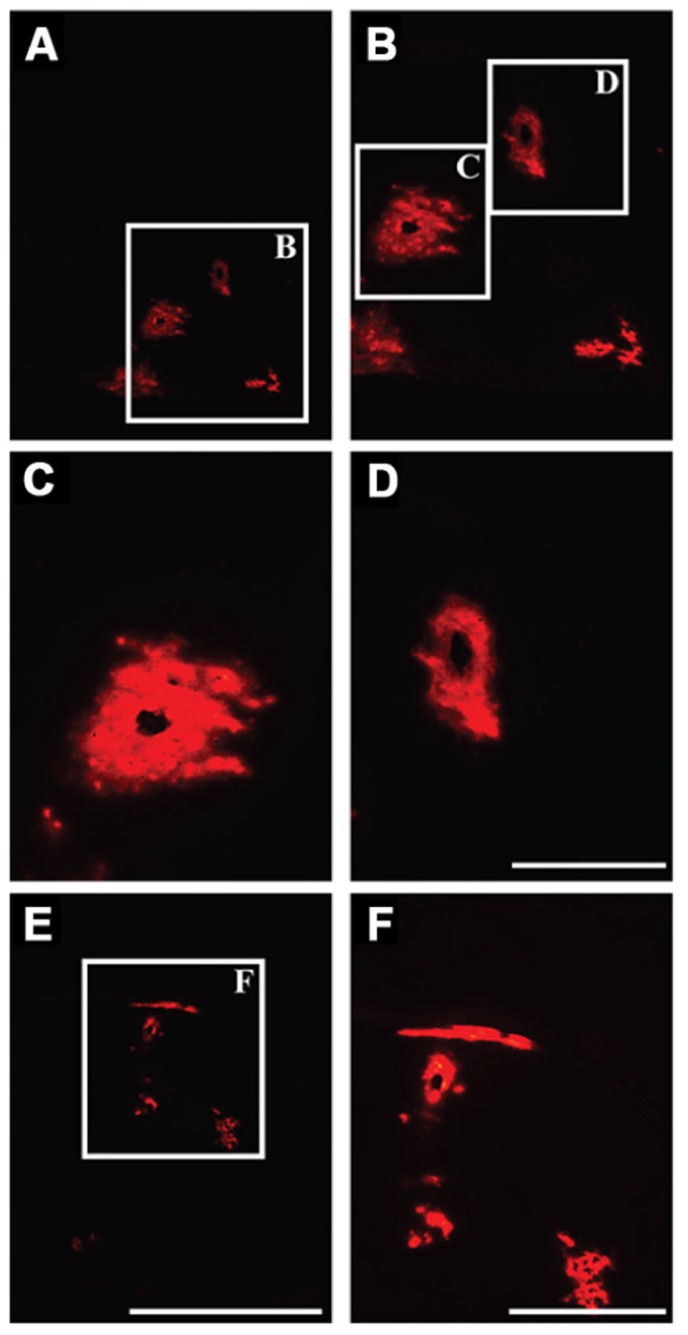
**Perivascular transport of liposomes. (A–F)** Examination of parenchymal tissue 2 mm rostral from the site of infusion showing the presence of fluorescently labeled liposomes surrounding the lumen of blood vessels, indicating effective transport of these particles along perivascular spaces. Scale bars: **A,E** = 5 mm; **C,D** = 500 μm; **B,F** = 1 mm.

**FIGURE 4:**
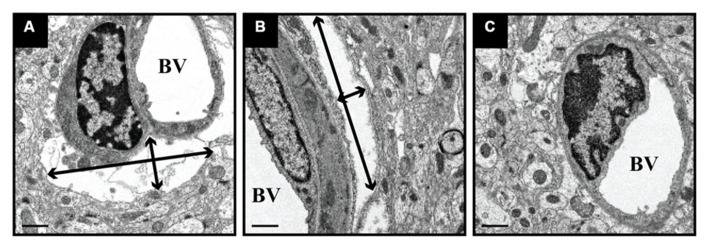
**Expansion of perivascular spaces after CED.** PBS-infused tissue was processed for EM and revealed the expansion of perivascular spaces after CED. These spaces ranged in size from 11 μm (width) × 3 μm (height; black arrows; **A**) and 2.0 μm (w) × 11.5 μm (h; **B**), as compared to non-infused, naïve control tissue **(C)**. BV = blood vessel lumen. Scale bar: 1 μm.

We delivered fluorescently labeled liposomes into the thalamus of adult rats and evaluated liposomal distribution in the brain 30 min after delivery. Interestingly, we observed liposomes approximately 2.4 mm rostral and 1.6 mm caudal from the site of injection (**Figure 2**). Given that the adult rat brain is approximately 20 mm in length along the anterior–posterior (AP) axis (excluding the olfactory bulb; Paxinos and Watson, 1998), we estimate that these liposomes efficiently traveled up to 4 mm on the AP axis, covering 20% of the rat brain. Consistent with our previous observations, we found fluorescently labeled particles surrounding blood vessels (**Figure 3**), indicative of perivascular transport. Furthermore, examination of the macro-environment by EM, revealed the expansion of the extracellular-perivascular space compared to normal non-infused control tissue (**Figure 4**). Margin measurements of these pockets revealed that the hydrostatic pressure of CED expanded the interstitial (Virchow–Robin) space sufficient to accommodate the distribution of large nanoparticles.

### CEREBROSPINAL FLUID (CSF) IS AN EFFICIENT DISTRIBUTOR OF BOTH AAV AND MICELLES

**FIGURE 5:**
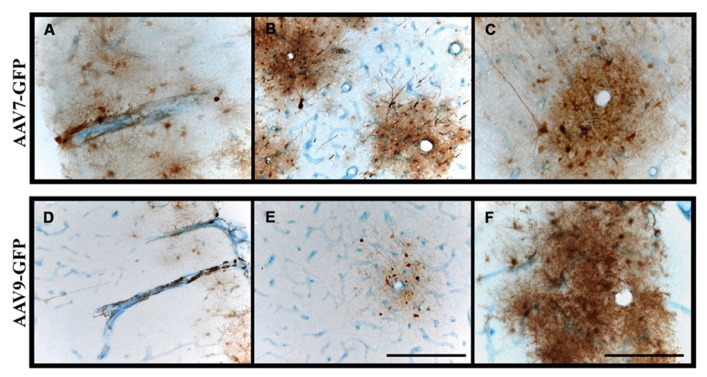
**Perivascular transport and pattern of transgene expression. (A–F)** Immunostaining against GFP and blood vessels (CD31; endothelial marker) revealed the presence of GFP-positive cells surrounding blood vessels. Transgene expression was frequently observed to form rosette-like patterns in white matter, as demonstrated in cross-sectional views of blood vessels **(B,C,E,F)**, indicative of perivascular transport of AAV particles irrespective of the serotype. Scale bars: **A,B,D,E** = 500 μm; **C,F** = 250 μm.

A third means by which nanoparticles and viruses may be distributed efficiently throughout the brain is via CSF. In previous studies, we found that intrathecal (cisterna magna) administration of AAV7 or AAV9 in NHP resulted in extensive transduction of neuronal and non-neuronal cells throughout the spinal cord, brainstem and neocortex (Samaranch et al., 2013). The pattern of distribution throughout white matter was non-uniform and took the form of rosettes that appeared to surround blood vessels indicated by co-staining against the endothelial marker, CD31, and GFP transgene (**Figure 5**). In many cases, the lumen of the vessel is patent and surrounded by endothelium stained blue and brown GFP^+^ cells (mostly astrocytes). This pattern is consistent with the concept of para-arterial flow of CSF through the brain (Nedergaard, 2013) and indicates that brisk flow of CSF from choroid plexus offers efficient vectorial flow through the cortex and cerebellum, as well as into the dorsoventral axis of the spine. Little transduction of subcortical gray matter is evident in these experiments, suggesting that binding of AAV7 and AAV9 to perivascular cells is relatively rapid, and the lack of transduction in deeper subcortical structures is a consequence of substantial sequestration of vector particles from superficial cortical surfaces first bathed by the CSF.

**FIGURE 6:**
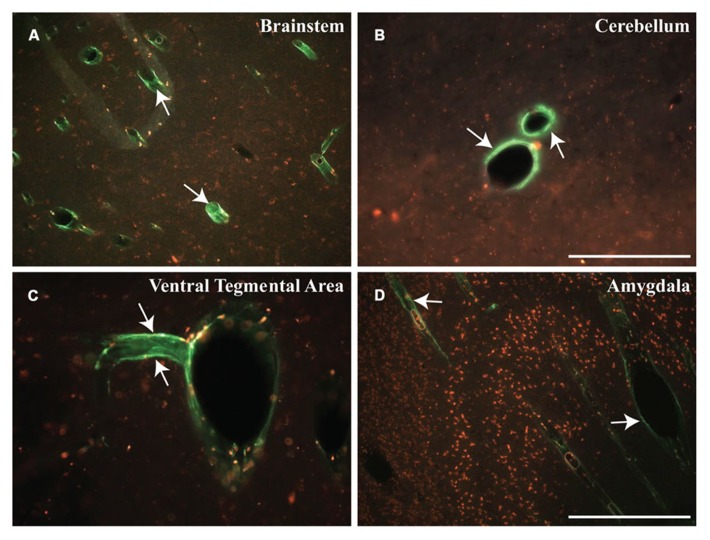
**Early distribution of micelles. (A–D)** Cisternal injection of fluorescently labeled micelles (shown in green) indicate the rapid transport of these particles within different regions of the NHP brain. These micelles filled the perivascular space of blood vessels throughout the brain, particularly in the brainstem (BS), cerebellum (CB), ventral tegmental area (VTA), and amygdala (AM). Note that these micelles are shown in green (white arrows) and presence of autofluorescent lipofuscin is shown in red. Scale bars: **A,D** = 500 μm; **B,C** = 250 μm.

Distribution of relatively inert nanoparticles, demonstrated that CSF can easily distribute infusates into the cortical gray matter. Recently developed ultra-small (~15 nm in size) spherical micelles (Dong et al., 2012), called “3-helix micelles,” based on amphiphilic peptide-PEG conjugates where the head-group self-associates into a 3-helix bundle, were covalently labeled with OG and infused into the CM of a *Rhesus* monkey. As shown in **Figure 6**, fluorescent nanoparticles shown in green, filled the perivascular space surrounding blood vessels 4 h after cisternal injection of 18 mg micelles in a 3 mL injection. In contrast to what we observed with AAV, where transduction was confined to white matter tracts, brainstem, cerebellum and spinal cord, infusion of micelles generated prominent perivascular fluorescence not only in brainstem and cerebellum, but also in the ventral tegmental area (VTA) and amygdala.

## DISCUSSION

Effective therapies for neurological diseases must take account of the profound anatomical and functional complexity of the brain. This is doubly so for directly delivered therapies. In this study, we demonstrated the distribution of nanoparticles of different sizes, including micelles (~15 nm in size), AAV (~20–25 nm) and liposomes (~65 nm), within the CNS of rodents and NHPs. Early work in understanding the distribution of AAV infused under pressure into the brain parenchyma revealed that the primary means of distributing vector was via perivascular (Virchow–Robin) spaces through which CSF flows. It is important to note that simple injections cannot hope to engage the perivascular system. Specialized infusion cannulae are required that enable constant pressures to be exerted at the tip of the cannula such that the interstitial hydrostatic pressure is exceeded and infusate can flow out into the tissue. Simple needles generate significant reflux and we introduced a reflux-resistant cannula to counter this tendency (Krauze et al., 2005c; Varenika et al., 2009). The advent of MRI-guided infusions further refined our understanding of the mechanics of perivascular flow and we showed in a number of studies that perivascular distribution of liposomes was linear with respect to time (Krauze et al., 2005a, 2006, 2009); the slope of the curve was increased in myelinated regions (Krauze et al., 2005b) and cessation of infusion prevented further expansion in the volume of distribution. Moreover, cannula placement is an important variable due to leakage of infusate into adjacent ventricles and/or white matter tracts (Yin et al., 2009, 2010a,b). No further increase in volume of distribution is seen once significant leakage occurs, presumably because of a sharp reduction in infusion pressure. In addition to a loss in infusion pressure, leakage of infusate into the ventricular system will lead to unrestricted exposure of the delivered agent within the CNS. Similarly, white matter tracts will act as a conduit and greatly distribute infusates throughout, ideal when attempting global exposure but undesirable when delivering therapeutics to localized regions.

This requirement for a pressure gradient in parenchymal delivery is not essential in the delivery of nanoparticles into CSF. The rate of flow of CSF is so high and the compartment so compliant that injections into CSF of quite large relative volumes do not cause significant increases in intracranial pressure in primates. We routinely inject up to 6 mL into either CM or lumbar intrathecal space without complications. The pace of CSF flow acts as a remarkably effective carrier for nanoparticles such as AAV or micelles. However, because this approach has remained relatively unexplored until recently for gene therapy (Snyder et al., 2011; Federici et al., 2012; Samaranch et al., 2013), the transduction properties of different AAV serotypes in primates is still unclear. The rosettes of transduction around blood vessels that we see with AAV7 and AAV9 result primarily from transduction of perivascular astrocytes. On that basis, one would predict that a highly neurotropic virus such as AAV2 would be poorly effective at transducing cells via the CSF route. Conversely, vectors that transduce astrocytes would be expected to be more efficient at CSF-mediated transduction of the brain. This, however, remains to be elucidated. It is clear, also, from infusion of non-viral nanoparticles that rapid distribution of CSF infusates is an inherently efficient process. In addition to distribution of fluorescent micelles throughout cerebellum, brainstem and cortex, we saw quantitative distribution into the amygdala and VTA, which provides encouragement to the idea that chronic drug delivery to the brain could be mediated by CSF infusion of drug-loaded micelles.

Axonal transport of AAV has turned out to be an important discovery. It reminds us forcefully that transduction patterns in the primate brain are strongly influenced by neuroanatomy and that neuronal projections act as efficient conduits through which intact virions may be transported. Indeed, this same propensity for neurons to transport other viruses, such as HSV (Diefenbach et al., 2008), is well-established. In fact, this phenomenon has been proposed as a means by which neurological diseases such as Alzheimer’s (Balin et al., 1998, 2008) and Parkinson’s disease (Braak et al., 2003) display their characteristic anatomical progression. Thus, it has been proposed that pathogens gain access to the brain via peripheral neurons and then steadily progress through interconnected regions. The Braak hypothesis of Parkinson’s disease, for example, is that the vagal nerve serves as a primary conduit through which a pathogen could work its way up through the brainstem to the midbrain and then to cortical regions (Braak and Braak, 2000). This anatomical progression is perfectly consistent with staging and progression of the disease where lesions in specific nuclei give rise to stage-specific symptoms, such as orthostatic hypotension as sympathetic innervation to the heart atrophies as parkinsonian degeneration attacks the brainstem. Relevant to the data shown here, we speculate that, even if disease-compromised neurons were not able to transport therapeutic agents to the region-of-interest, an alternative approach would be to design agents that could be easily transported via the perivascular space, particularly when trying to reach regions of motor control such as the brainstem.

The *in vivo* distribution of nanoparticles within the cerebral cortex, as shown here, invokes certain considerations for gene and drug therapy. For instance, liposomes, almost three times larger in size than an AAV particle, efficiently covered 20% of the rat’s brain within 30 min after delivery, indicating the synergistic potential of delivering agents through a pressurized system to engage with the pulsating parenchyma to enhance the amount of brain volume covered by the agent. AAV, liposomes and micelles are essentially nanocarriers designed to deliver therapeutic payloads and in a clinical setting elevation or reduction of a patient’s heart rate during anesthesia, could play a significant role in the distribution of therapeutic agents (i.e., at least in those delivered within the parenchyma). Obviously, this requires further investigation.

In conclusion, advances in biomaterials and development of nanoparticles are enabling a better understanding of the complex functionality of the central nervous system. Here we describe the movement of nanoparticles of different composition and size within the brain of rodents and NHPs. Tools such as fluorescent reporter proteins in AAV and fluorophore-conjugated nanoparticles have allowed us to better understand some key aspects associated with perivascular transport, CSF flow and axonal transport. However, even though it is unclear how these routes of transport would be affected in a diseased-brain, the diverse tools we have in hand should continue to advance our knowledge in this area.

## AUTHOR CONTRIBUTIONS

Ernesto A. Salegio, Hillary Streeter, Nikhil Dube, Lluis Samaranch, Adrian P. Kells, Waldy San Sebastian, John Forsayeth, Krystof S. Bankiewicz helped design and conduct the rodent experiments. Ernesto A. Salegio, Hillary Streeter, Lluis Samaranch, Adrian P. Kells, Waldy San Sebastian, Krystof S. Bankiewicz helped with NHP surgeries, daily care of animals and tissue processing. Nikhil Dube, Ting Xu made the micelles and helped with data analysis. All authors contributed to the writing of the manuscript.

## Conflict of Interest Statement

The authors declare that the research was conducted in the absence of any commercial or financial relationships that could be construed as a potential conflict of interest.
